# Introduction to the Special Issue on Early Child Development: From Measurement to Optimal Functioning and Evidence-Based Policy

**DOI:** 10.3390/ijerph18105154

**Published:** 2021-05-13

**Authors:** Verónica Schiariti

**Affiliations:** Division of Medical Sciences, University of Victoria, Victoria, BC V8W 2Y2, Canada; vschiariti@uvic.ca; Tel.: +1-250-472-5500

##  *The Importance of Early Child Development* 

Early child development and overall children’s developmental trajectories have long-term implications for health, functioning, and earning potential as these children become adults. Importantly, failing to reach developmental potential contributes to the global cycles of poverty, inequality, and social exclusion. The recognition of the close connection between the development of the child and the development of families and communities has served as the basis for national and global initiatives in health and education in recent decades.

In 2015, for the first time, an international initiative—the 2030 Sustainable Development Goals (SDGs)—included an early childhood development goal. Specifically, SDG 4 is to ensure lifelong learning, early stimulation, increased length of schooling, school performance, and adult income. In addition, early childhood development is intricately linked to other SDG goals, poverty reduction, health and nutrition, equality for girls and women, and ending violence. Nevertheless, the risks for delay, disability and unmet potential in the early development of young children are still pervasive, particularly in low- and middle-income countries.

This editorial letter introduces the main contribution of this Special Issue and invites the readers to explore and share its content to benefit a wider audience. The Special Issue is dedicated to Early Child Development: from measurement to optimal functioning and evidence-based policy, it includes 15 open access papers addressing key topics of global interest and application.

##  *From Measurement to Optimal Functioning and Evidence-Based Policy* 

Early child development measurement tools and their association with long-term outcomes were covered by many papers included in this issue [1,2,3,4,5]. Janus et al. [1] synthesized research using the Early Development Instrument (EDI), which was developed in Canada as a population-level assessment of children’s developmental health at school entry. This narrative review shows the ability of the EDI to monitor children’s developmental outcomes in various populations, how the EDI contributes to expanding the understanding of the impacts of social determinants on child development, and how it applies to special populations. Moreover, Isquith-Dicker et al. [2] examined the associations between the data obtained using child development assessment tools and educational attainment, academic achievement, or wealth. Their review demonstrates the potential for certain child development assessment tools to adequately assess long-term outcomes, but identifies that additional prospective studies using validated, culturally appropriate tools are needed. On the other hand, Napoli et al. [4] developed a novel International Classification of Functioning, Disability and Health-based tool for children with autism spectrum disorder (ASD) in Argentina, called TEA-CIFunciona. This is the first ASD culturally sensitive pediatric tool that guides the comprehensive description of functioning in Latin America. In addition, Schneide et al. [5] describe the cross-cultural validation of the responsive interactions for learning measure, the authors show that the Brazilian Portuguese version is a valid and reliable instrument for a brief assessment of responsive caregiving.

Different developmental trajectories of specific areas such as language development [3], nationwide growth patterns [6] and vision development [7] were covered by the original papers conducted in South Korea [3,6] and Spain [7]. Interestingly, Pinero-Pinto et al. [7] present the first study in a large sample of toddlers, describing the developmental correlations between visual and motor systems in typical developing toddlers.

Enabling optimal functioning, with special emphasis on child–environment interactions during early childhood, was addressed by several papers in this Special Issue. For example, Bendini & Dinarte [8] from The World Bank explored the effect of maternal depression on early childhood cognition in Peru using data from the Young Lives Survey [8]. Their findings make a strong case for recognizing maternal mental health problems as disorders of public health significance and guide maternal and infant health policies in Peru.

A qualitative study led by van Zyl, C [9] explored the environmental modifiable factors that could potentially have affected the first 1000 days of absent learners in the Western Cape of South Africa. The authors found six predominant themes that played a role during the first 1000 days of the lives of these absent learners, including health and nutrition of both the mothers and their children, substance use/abuse during pregnancy, toxic stress, support received by the mothers and their children, attachment, attentive care, and stimulation and play.

Professor McWilliam R.A and his international collaborators from 10 countries contributed to a project report on the global implementation of the Routines-Based Model (RBM) [10], sharing implementation challenges and successes, the group concluded that support-based visits with families—an RBM hallmark—can shape excellent early intervention practices for international use.

A narrative review [11], co-authored by professor Simeonsson R., synthesizes key global initiatives driving the international agenda on early child development. Furthermore, this paper proposes a universal assessment and intervention framework promoting the developmental potential in early childhood to be adopted across sectors [11].

Lastly, a study protocol by Longo et al., entitled GO-ZIKA-GO, describes a promising child-friendly environmental intervention for children affected by congenital Zika syndrome in Brazil [12].

A special mention to the paper led by Yu Hsin et al. [13], a collaboration between Sweden, U.S.A, and Dubai, which investigates the impact of using assistive technology (video-coding approach) to facilitate communication and social participation for all children with complex needs in day-to-day functioning. As such, the topic of this study enhances the content of this Special Issue, particularly how to promote optimal functioning in children and youths who need it the most. The study advocates for children’s rights to express their opinions regardless of their physical limitations.

The implication of contributing quality early child development data that can ultimately guide evidence based-policy was covered by many papers [8,9,11,13,14,15]. Notably, Gladstone et al. [14] report on the psychometric properties of the World Health Organization (WHO) indicators of Infant and Young Child Development (IYCD). Specifically, the study validates the IYCD in Brazil, Malawi and Pakistan, showing that the IYCD performed well in cognitive testing, had similar developmental trajectories and high reliability across countries. Importantly, the IYCD initiative creates a set of global population-based developmental indicators for the currently overlooked population of children aged 0 to 3 years of age, a major contribution that will provide data/evidence of the developmental status and needs of this population, a crucial step for creating evidence-based polices globally.

Finally, a paper by Schiariti & McWilliam [15] focuses on innovative strategies promoting collaborative empathic teleintervention for children with disabilities during and post the COVID-19 lockdown. This perspective paper proposes to apply principles of RBM beyond the age of five in combination with novel rights and ability-oriented approach e-tools—e.g., My Abilities First [15].

##  *Contributions of This Special Issue to the International Community* 

This Special Issue raises global awareness of the importance of children’s first years of life and the crucial role of child–environment interactions where the child lives, plays, and grows. The papers included in this Special Issue represent all the continents with diverse cultural and socioeconomic backgrounds. International experts generously shared information related to pivotal topics in early child development, including the need of culturally sensitive tools, successful transdisciplinary models of care, novel intersectoral frameworks, population-based data, and ongoing and upcoming environmental projects that invite positive change in practices. As such, this Special Issue contributes—to the global community—novel information promoting optimal functioning in natural environments, ultimately guiding high-quality programs and evidence-based policies for young children around the world.

## Data Availability

Data sharing is not applicable to this article as no new data were created in this editorial. The data supporting the author’s comments are available within the reference list.

## References

[1] Janus M., Reid-Westoby C., Raiter N., Forer B., Guhn M. (2021). Population-Level Data on Child Development at School Entry Reflecting Social Determinants of Health: A Narrative Review of Studies Using the Early Development Instrument. Int. J. Environ. Res. Public Health.

[2] Isquith-Dicker L., Kwist A., Black D., Hawes S., Slyker J., Bergquist S., Martin-Herz S. (2021). Early Child Development Assessments and Their Associations with Long-Term Academic and Economic Outcomes: A Systematic Review. Int. J. Environ. Res. Public Health.

[3] Park E.-Y. (2021). Stability of the Communication Function Classification System among Children with Cerebral Palsy in South Korea. Int. J. Environ. Res. Public Health.

[4] Napoli S., Vitale M., Cafiero P., Micheletti M., Bradichansky P., Lejarraga C., Urinovsky M., Escalante A., Rodriguez E., Schiariti V. (2021). Developing a Culturally Sensitive ICF-Based Tool to Describe Functioning of Children with Autism Spectrum Disorder: TEA-CIFunciona Version 1.0 Pilot Study. Int. J. Environ. Res. Public Health.

[5] Schneider A., Rodrigues M., Falenchuk O., Munhoz T., Barros A., Murray J., Domingues M., Jenkins J. (2021). Cross-Cultural Adaptation and Validation of the Brazilian Portuguese Version of an Observational Measure for Parent–Child Responsive Caregiving. Int. J. Environ. Res. Public Health.

[6] Yoon S., Lim J., Han J., Shin J., Lee S., Eun H., Park M., Park K. (2021). Identification of Growth Patterns in Low Birth Weight Infants from Birth to 5 Years of Age: Nationwide Korean Cohort Study. Int. J. Environ. Res. Public Health.

[7] Pinero-Pinto E., Pérez-Cabezas V., De-Hita-Cantalejo C., Ruiz-Molinero C., Gutiérrez-Sánchez E., Jiménez-Rejano J.-J., Sánchez-González J.-M., Sánchez-González M.C. (2020). Vision Development Differences between Slow and Fast Motor Development in Typical Developing Toddlers: A Cross-Sectional Study. Int. J. Environ. Res. Public Health.

[8] Bendini M., Dinarte L. (2020). Does Maternal Depression Undermine Childhood Cognitive Development? Evidence from the Young Lives Survey in Peru. Int. J. Environ. Res. Public Health.

[9] van Zyl C., van Wyk C. (2021). Exploring Factors That Could Potentially Have Affected the First 1000 Days of Absent Learners in South Africa: A Qualitative Study. Int. J. Environ. Res. Public Health.

[10] McWilliam R.A., Boavida T., Bull K., Cañadas M., Hwang A.-W., Józefacka N., Lim H.H., Pedernera M., Sergnese T., Woodward J. (2020). The Routines-Based Model Internationally Implemented. Int. J. Environ. Res. Public Health.

[11] Schiariti V., Simeonsson R.J., Hall K. (2021). Promoting Developmental Potential in Early Childhood: A Global Framework for Health and Education. Int. J. Environ. Res. Public Health.

[12] Longo E., De Campos A.C., Barreto A.S., Coutinho D.L.D.L.N., Coelho M.L.G., Corsi C., Monteiro K.S., Logan S.W. (2020). Go Zika Go: A Feasibility Protocol of a Modified Ride-on Car Intervention for Children with Congenital Zika Syndrome in Brazil. Int. J. Environ. Res. Public Health.

[13] Hsieh Y.-H., Borgestig M., Gopalarao D., McGowan J., Granlund M., Hwang A.-W., Hemmingsson H. (2021). Communicative Interaction with and without Eye-Gaze Technology between Children and Youths with Complex Needs and Their Communication Partners. Int. J. Environ. Res. Public Health.

[14] Gladstone M., Lancaster G., McCray G., Cavallera V., Dua T., Lindgren Alves C.R., Malawichi L., Rasheed M., Janus M., Karige P. (2021). Validation of the W.H.O Indicators of Infant and Young Child Development (IYCD) in three countries; Brazil, Malawi and Pakistan. Int. J. Environ. Res. Public Health.

[15] Schiariti V., McWilliam R.A. (2021). Crisis Brings Innovative Strategies: Collaborative Empathic Teleintervention for Children with Disabilities during the COVID-19 Lockdown. Int. J. Environ. Res. Public Health.

